# Environmental and Economic Viability of Using Concrete Block Wastes from a Concrete Production Plant as Recycled Coarse Aggregates

**DOI:** 10.3390/ma17071560

**Published:** 2024-03-28

**Authors:** Jorge Los Santos-Ortega, Esteban Fraile-García, Javier Ferreiro-Cabello

**Affiliations:** Department of Mechanical Engineering, Mechanical Area of Media Continuous and Theory of Structures, University of La Rioja, 26004 Logroño, Spain; esteban.fraile@unirioja.es (E.F.-G.); javier.ferreiro@unirioja.es (J.F.-C.)

**Keywords:** recycled coarse aggregates, natural coarse aggregates, concrete, concrete block waste, construction and demolition waste, sustainability, life cycle assessment

## Abstract

The construction sector must incorporate the circular economy to improve sustainability and efficiency. The use of recycled aggregates (RAs) as a substitute for natural aggregates (NAs) is currently being investigated and is expected to yield considerable benefits in the future. The objective of this research is to evaluate the environmental and economic benefits of using recycled coarse aggregates (RCAs) in different 1 m^3^ samples of concrete, substituting the natural coarse aggregate (NCAs) with RCAs in different percentages. RCAs generally come from the treatment of construction and demolition wastes (CDWs). However, in this research, the RCAs are the concrete block wastes (CBWs) generated by a concrete production plant. Among the most notable results is that compared to concrete with no RCAs, using alternatives in which RCAs have replaced 50% of the NCAs leads to an average decrease in impact category statistics of −3.30%. In contrast to the existing literature on the subject, the process of producing RCAs generated efficiency improvements in categories such as abiotic depletion of fossil fuels (−58.72%) and global warming potential (−85.13%). This is because the transport process, a key factor in determining the viability of using RAs instead of NAs, was eliminated. In economic terms, there is a slight decrease in the financial cost of producing 1 m^3^ of concrete as the quantity of RCAs increases. The maximum decrease was 0.23€/m^3^ in the samples studied. Combining both the environmental and economic aspects resulted in a reduction factor of 0.420 g of CO_2_/€cent, which means fewer CO_2_ emissions per unit cost when using RCAs. In conclusion, these results are intended to further knowledge in the field of using RAs instead of NAs in order to help the sector achieve sustainability and find an alternative use for a particular type of business waste.

## 1. Introduction

The construction sector, and within it, the sub-sector dedicated to concrete production, is facing a new challenge. The aim is to achieve sustainability and thus develop cleaner and more efficient production overall, not only for the environment but also for society and human health. To this end, the circular economy is key to achieving this much-needed sustainability. Fundamentally, transitioning to a circular economy involves reincorporating waste into the value chain and, in doing so, converting something that has been considered unusable for decades into new raw materials that can be used in future products [1]. As a result, governments and public administrations are already working on new construction sector regulations [2] that will allow waste to be incorporated into products, without prejudice, at any point, to the minimum safety conditions that are so important in this industry. Concrete and mortar are the materials par excellence in the construction sector. These two materials offer an excellent opportunity to apply circular economy principles because, generally speaking, they are composed of cement, fine or coarse aggregates and water. The conventional literature on this subject already contains examples of studies and research into the complete or partial substitution of compounds such as cement and aggregates (fine and coarse) with a new element obtained from waste [3,4,5,6,7].

The use of recycled aggregates (RAs) is being successfully introduced in various construction activities. Their use is mainly intended to reduce the exploitation of natural resources and the generation of waste, which mainly ends up in landfill [8]. However, using RAs may lead to higher CO_2_ emissions and energy consumption due to the upstream recycling process required to prepare them. Among the most popular and well-known RAs are those that originate from the process of recycling construction and demolition wastes (CDWs) [9]. In Europe, it is regarded as one of the most significant sources of waste, accounting for 25~30% of all waste [9]. The volume of CDW production makes this waste a priority for treatment. As a result of the interest in this waste, increasing research is being carried out on the use of CDWs as RAs and their application in the construction sector. For example, in the field of pre-cast concrete, the research by López et al. (2016) [8], evaluates the possibility of using RAs originating from CDWs as aggregates in concrete kerbs. One of the study’s most notable findings was that substituting 50% of the natural aggregates (NAs) with RAs results in a 5.78% increase in energy consumption and, therefore, in the emission of pollutants. They calculated an increase in CO_2_ emissions of 67.3 t in one year [8]. These increased environmental impacts are explained, as mentioned above, taking into account that the CDWs must be treated to obtain the RAs. This implies the need for additional processes, such as transport, crushing, and sorting, which logically add to the number of operations required to produce the RAs, and, in turn, means that the associated environmental impacts are greater than those associated with producing NAs. This increase in environmental impacts has already been identified by other authors [10,11,12,13].

So, what environmental advantage is gained by using RAs instead of NAs? The answer lies in the environmental impact categories directly related to the production of NAs, such as depletion of natural resources, land occupation, water depletion, and avoidance of landfill. This is evidenced by Pu et al. (2023) [14], who found that replacing 100% of NAs with RAs decreases the impact categories of acidification by 20.1%, Photochemical oxidant creation by 5.4%, and eutrophication by 15.11% [14]. Not only do RAs help to reduce certain environmental impact categories, but also categories related to human health. These are detailed in the research by Cerchione et al. (2023) [13], who identified decreases of 27.1% for the human carcinogenic toxicity category and 77.9% for the human non-carcinogenic toxicity category when NAs are replaced by RAs [13]. These categories, however, are less well known by society at large because discussions of environmental impacts often focus entirely on impact categories such as CO_2_ emissions and fossil fuel depletion [15].

As mentioned above, RAs can come from various sources, although the vast majority come from CDWs because of the interest in finding an alternative use for them. Other RAs can come from the waste generated by a company’s in-house production. This is the case in this research, which uses the in-house concrete block wastes (CBWs) produced by the concrete manufacturer Hormigones Ebro (Navarra, Spain). The CBWs can be recycled within the concrete production plant itself to obtain recycled coarse aggregates (RCAs), and once obtained, reused as a substitute for natural coarse aggregates (NCAs). The physical-mechanical and chemical properties of these RCAs, as well as their behaviour in concrete samples, have already been evaluated [16]. Their results show that these RCAs possess the minimum conditions required for their use according to the current structural regulations [2]. However, the mechanical results showed a slight decrease in the tensile strength, and a greater dispersion in the results derived from a higher heterogeneity of the mixtures. An expected phenomenon when using RCAs instead of NCAs [16]. As may be expected, this type of waste may have advantages over RCAs made from CDWs. The CBWs do not have to be transported as they are recycled within the plant itself. Consequently, a new gap in the literature is identified, as it is possible to evaluate how the viability of these RCAs increases in the absence of a prior transport process. This, as will be shown later, represents a considerable advantage. In addition, this type of concrete waste is more homogeneous than CDWs as it does not contain impurities such as metal waste, bricks, and plastics, which hinder the recycling process [17]. The use of concrete waste has already been highlighted by other current research, which also proposes the use of this type of waste as aggregates, as is the case of research by Revilla et al. (2022) [18], or Trinchese et al. (2022) [19]. However, this research focuses only on characterising the behaviour of recycled concrete aggregates in a matrix, and does not generate results on their environmental and economic sustainability. This is a knowledge gap, which in the case of this research will be filled with a case study of the RCAs, and a comparison between them and the current literature on RCAs.

These antecedents, which suggest a positive future for the use of RAs over NAs, provide the basis for this research. Its objective is to carry out an environmental assessment of the reuse of CBWs from a concrete manufacturing plant using life cycle assessment (LCA) methodologies. Based on the results obtained in the previous research, the second objective is to determine the existence of the environmental and economic benefits of using RCAs in concrete. In order to determine its sustainability and thus be able to complement and add value to the previous results. Once this first part of research has been completed, and given the unique nature of this waste (produced internally within a concrete manufacturing plant), an environmental and economic comparison is made with the RCAs traditionally sourced from CDWs and studied in existing research in this field of study. With this discussion and comparison with other RCAs from different sources, the aim is to determine any existing differences and justify them. This will globally generate knowledge in the field of research on the use of RAs on NAs and provide results to achieve sustainability in the sector.

## 2. Materials

### 2.1. Cement

The cement used to prepare the samples was a conventional Portland Type I cement, designated as CEM I 32.5R according to the standards [20]. This type of cement contains a high percentage of clinker of between 95~100%, and its characteristic compressive strength is 32.5 MPa at 28 days of age. This type of cement is used to make conventional concretes.

### 2.2. Natural Fines and Coarse Aggregates

The fine aggregates and NCAs used in the research come from a local quarry owned by Hormigones Ebro, a collaborator in this research. The fine aggregates used were of type FA-0/6-T-S-L according to the designation set by the regulations [2]. A fine aggregate with a particle size of 0–6 mm was used. Two particle sizes were used for the coarse aggregates. The first was a 6–12 mm GR-6/12-T-S-L aggregate, and the second was a larger aggregate of 12–20 mm called GR-12/20-T-S-L. The aggregates correspond to those typically used for the production of conventional concrete at the concrete production plant [16].

### 2.3. Recycled Coarse Aggregates (RCAs)

The RCAs were made from the CBWs generated by the Hormigones Ebro concrete production plant. It offers a way of recycling the company’s in-house production waste and may also be applicable to pre-cast concrete companies. The RCAs obtained are called CA-12/20-T-R according to the standard [2]. In general, RCAs should be studied prior to their application in concrete. The aim is to characterise its physical-mechanical and chemical properties according to the conditions established by current structural regulations. In the case of this research, these RCAs were already defined and studied [16], with the conclusion that they may be viable for use in concrete according to current regulations in Spain [2].

### 2.4. Water

The water used was industrial water obtained directly from the mains. It did not undergo any kind of pre-treatment.

### 2.5. Concrete Mixes

Samples containing different proportions of cement, NAs, RCAs and water were mixed to make concrete. Their raw material quantities are shown in Table 1. RCAs were substituted at several percentage levels, namely 15%, 20%, 30% and 50%. These substitution percentages correspond to the weight of NCAs replaced by RCAs. Each sample was coded as ‘H-x’, where ‘x’ is the percentage of NA substituted by RA in weight (kg). Sample H15, for example, represents a sample where 15% by weight of the NCA (CA-12/20-T-R) was replaced by RCA type CA-12/20-T-R.

## 3. Methodology and Case of Study

This section describes the methodological techniques used in the research. An environmental methodology was applied through the life cycle assessment (LCA) procedure, allowing the identification and quantification of the environmental impacts that occur in relation to a process, product or service. This aims to assess the viability of using the RCAs in this case study, where these new RCAs come from a different source than traditional RCAs obtained from the recycling process of CDWs, as will be explained in detail in subsequent sections. To these environmental results, which gather the effects on various elements such as soil, water and atmosphere, economic results are added in order to determine economic viability. To analyse the economic viability, the prices comprising the materials for the production of 1 m^3^ of concrete will be studied, and therefore, depending on the percentage of substitution, it will be determined whether there is an economic decrease. This new economic viability, together with the mechanical factors of RCAs are key variables for their use in applications. In doing so we have sought to provide knowledge and take a different approach to previous research [16], which, conventionally, in this field of study, only evaluates the mechanical performance of RCAs used in concrete. In the following sections, both methodologies will be described more concisely, detailing their respective stages before the final results are obtained.

### 3.1. Life Cycle Assessment (LCA)

As introduced above, the LCA tool is now a widely and increasingly used technique. It is essential for understanding and promoting the sustainability of the various sectors that make up the industry and is applied in this research to the construction sector. Basically, this methodology allows the environmental aspects associated with a product or service to be assessed throughout its life cycle (or part of its life cycle). Rather than being freely applicable, this methodology is regulated by the UNE-EN ISO 14040 [21] and UNE-EN ISO 14044 [22] standards, which establish a series of intermediate stages to structure the methodology and achieve the objective of obtaining environmental results for the element that is to be evaluated. These intermediate stages are detailed below.

#### 3.1.1. Objective and Scope

The main objective is to determine the environmental viability of using RCAs made from the waste generated by a concrete production plant. Specifically, the CBWs produced in the concrete production plant itself. The scope defined for this research is from cradle-to-gate for each of the different functional units (FU) that have been set.

#### 3.1.2. Functional Unit

Two functional units were used in this research. The first functional unit is 1 m^3^ of ready-mixed concrete. With this FU, the aim is to understand in a volumetric unit widely used in the construction sector [14,23] how the various environmental impacts are distributed among the material composites that make up the concrete. In other words, which raw material contributes the most negative or positive effect to the overall mixture, in addition to assessing the environmental suitability of the RCAs in the samples. This provides an overview of the picture as a whole and also allows conclusions to be drawn as to which elements are most harmful, with the aim of implementing new amelioration or mitigation techniques. A second functional unit of 1 tonne of RCAs was proposed [9,13,24] to conduct an in-depth study of the environmental differences between the use of NAs and RAs. This comparison will help us analyse exactly what the differences are between producing one tonne of NCA and one tonne of RCA. The environmental results of both FUs will reveal whether it is environmentally viable, on the one hand, to use RCAs instead of NCAs and, more specifically, whether using RCAs in concrete for the construction sector is environmentally cost-effective.

#### 3.1.3. System Boundary

The system boundary defines what is included in the study. Defining them is important because by limiting the system boundary, we can exclude various processes that affect the FU throughout its life cycle (cradle-to-grave), some of which may have considerable environmental impact [15]. Therefore, the system boundary set for an experiment must be well justified, as reducing the scope of the study could lead to erroneous results because of the reasons mentioned above. For this study, we decided to limit the system boundary to cradle-to-gate for 1 m^3^ of ready-mixed concrete. This is justified since it is in these early stages of raw material extraction and manufacturing that the greatest environmental impacts are generated, and therefore the greatest differences may exist between one alternative and another [25]. Subsequent phases of construction (A4–A5), use and maintenance (B1–B7), demolition and recycling (C1–C4) do have an impact on the environment, but these phases are currently assumed to be similar between the concrete alternatives assessed and, for that reason, they were not included in this research [14,15,26]. Existing literature on research into waste utilisation in building elements such as mortar and concrete also defines the system boundary as from cradle-to-gate [13,14,24,27].

**Figure 1:**
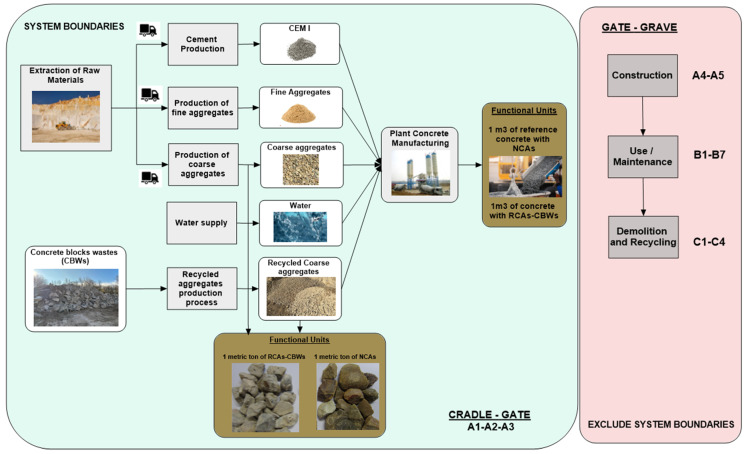
System Boundary for the study of concrete production with RCAs and CBWs. The grey boxes represent a process, the white boxes a material or waste, the brown boxes the functional unit.

Figure 1 shows the system boundary for the research and the processes that take place after the creation of the functional unit that are excluded from this research. The phases include the process of extracting the raw materials (A1) needed to make the concrete product (A3), in addition to their internal transport (A2). Figure 1 also shows the two FUs defined for this research. The first of these is 1 m^3^ of concrete, which will allow us to assess the various alternatives by varying the percentage of NCAs substituted with RCAs. The second FU is 1 tonne of RCA, which will allow us to compare results with the existing literature and interpret any differences. With regard to obtaining the RCAs, a wealth of literature is available describing the resources required during the process [8,10,13,14,28]. RCAs primarily come from recycled CDWs. However, not all RCAs have the same origin, as in this case study, which uses RCAs from concrete block waste (CBWs), the recycling and procurement process of which is described below. For better understanding, the results are presented for two types of RCAs. RCA-CBWs (the type used in this case study) and RCA-CDWs (RCAs derived from CDWs and covered in the existing literature). The following sections explain the existing system boundary encompassing the production of the RCA-CBWs and RCA-CDWs.

- RCA-CBWs System Boundary Comparison

**Figure 2:**
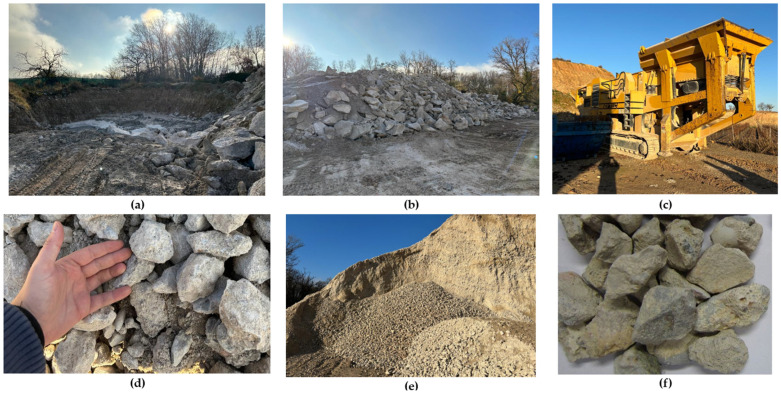
Production process for RCAs used in the research. (**a**) Pouring pit of fresh, dried concrete. (**b**) Broken up concrete blocks for recycling obtained from the pit. (**c**) Crushing machine for producing the RCA-CBWs. (**d**,**e**) Collection and size achieved by the initial crushing of the RCA-CBWs. (**f**) RCA-CBWs used in the research.

CBWs are made from the surplus or waste produced in the concrete plant itself. This waste is produced through the following process: residual fresh concrete that was not poured at the usage site is transported back to the plant in the concrete mixer trucks. This fresh concrete is poured into a pit, as shown in Figure 2. The fresh concrete is poured into the pit until reaches a certain level and is left to dry out in the open. Subsequently, an excavator with a hydraulic hammer demolishes and breaks up this residual concrete layer into large CBWs, as can be seen in Figure 2. Finally, these CBWs are fed into an aggregate recycling machine, where they are crushed to the size used in this research. Two sub-processes are used to crush the material to the appropriate aggregate size (see Figure 2).

**Figure 3:**
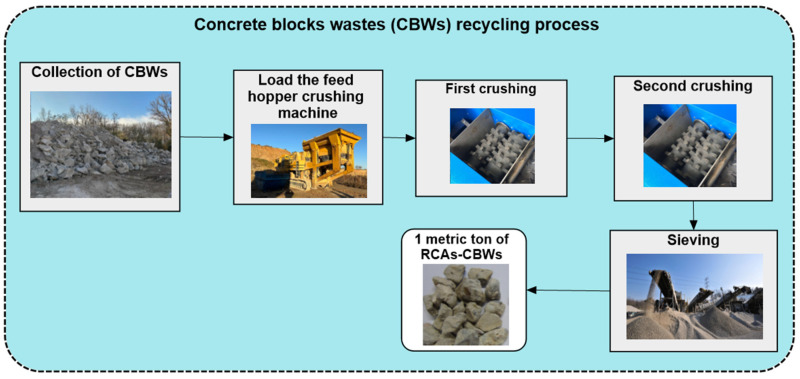
System Boundary for the CBW recycling process used in this case study.

Having explained the process of recycling concrete block waste, it should be noted that a very similar process is used to produce RCA-CDWs (as will be seen later), with the exception of a number of nuances. Firstly, CBWs do not need to be transported to the recycling plant, as the waste is collected within the plant itself, with negligible internal transport. Secondly, as CDWs do not contain any metallic elements, the crushed waste does not need to be passed through an electromagnet. In addition, CBWs have a more homogeneous composition, while CDWs come from the demolition of a building and the composition of the waste includes metals, ceramics, plastic, paint and tiles. Therefore, Figure 3 illustrates the system boundary for recycling CBWs to produce RCAs.

**Figure 4:**
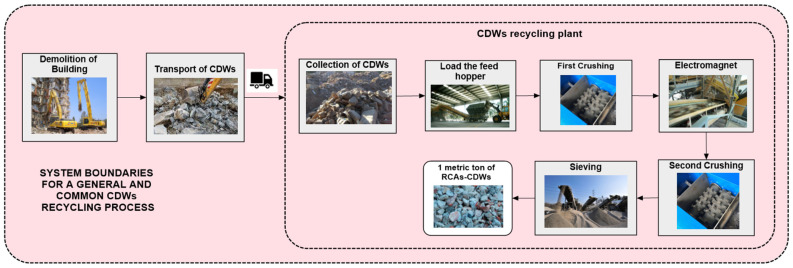
Recycling process of CDWs to obtain RCAs.

CDWs can be recycled in two types of plants. Mobile plants move to the place where a building is being demolished and, thus, the place where the CDWs are being produced. These plants treat the CDWs and produce the RCA-CDWs on-site. The treated product is then transported to the place where it will be used, such as a concrete production plant. The disadvantage of this type of plant is that its output is lower than that of a fixed plant, but because the recycling process takes place close to the supply of the CDWs, they do not have to be transported to the recycling plant, as is the case with fixed recycling plants. Fixed plants offer optimal recycling as they are usually more complex and can obtain RAs with different granulometries. They can also handle and store larger quantities of material, so their annual production rate is higher. The system boundary for the production of RCA-CDWs in a fixed recycling plant are shown generically in Figure 4.

The CDW recycling process starts with the demolition of the decommissioned building, which generates various waste materials that are loaded into a tanker truck and transported from the demolition site to the recycling plant. Transport is a key factor, as excessive distances can make recycling CDWs environmentally unfeasible or too costly. Once at the recycling plant, the raw material from the CDWs is stockpiled in large quantities. An excavator loads the feeding chute to ensure there is no lack of material supply in the process. The waste is first sprayed with water to prevent the emission of dust and particles during the process. Next, it is transported by conveyor belt for initial crushing by a toothed crushing machine. Subsequently, this reduced-sized waste is passed through an electromagnet to automatically extract the metal debris from the reinforcements and manually collect plastics and other undesirable waste. It is then subjected to secondary crushing in impact crushers to further reduce the size of the pieces. Lastly, it passes through a series of sieving processes that separate the RAs according to their granulometry and they are then stored in alluviums for later use. This is the simplest process used to obtain the RCA-CDWs, however, other methods obtain better quality aggregates, such as the heat method. This method removes the old mortar stuck to the aggregates. After the crushing processes, the RCAs are heated and filtered into aggregates and dust [14]. However, compared to the simple process described above, heat treatment uses a considerable amount of energy [14].

#### 3.1.4. Life Cycle Inventory (LCI)

The life cycle inventory (LCI) consists of compiling the quantities of materials, energy, waste, resources, etc., used in the processes included in the system boundary. Like the system boundary, the LCI is key to achieving consistent research results and therefore, special attention should be paid to ensuring it is as specific as possible. To create the LCI for this research, we used Hormigones Ebro’s own data to characterise the CBW recycling process. We referred to earlier research to improve the inventory of the process and compare it with similar processes. These were, firstly, the inventory provided by the National Association of Aggregates Manufacturers (ANEFA) (Madrid, Spain), which includes relevant data on the production of RCAs from CDWs in Spain in 2019 [17]. And, secondly, research on concrete case studies [13,24,29].

For this LCI, we had to divide the overall process into several sub-processes. These were the materials that make up the concrete such as NAs, cement and water. Subsequently, the consumptions involved in treating the CBWs to obtain the RCA-CBWs. And finally, the transport process applied to all the materials in order to model the environmental impacts of transporting them from their place of origin to their point of use. To model the LCA we used the SimaPro 9.2.0.2 software, entering the various quantities of materials, energy and waste identified in the LCI. SimaPro’s internal databases such as Ecoinvent v.3 [30] as well as the ELCD (European Life Cycle Database) [31] were used for this purpose.

- Materials

It begins by explaining the raw materials used in the samples evaluated. These are shown in Table 2, as well as the processes that were chosen in the SimaPro software and their respective database.

- Production process of RCA-CBWs

The process used to treat the CBWs generated by Hormigones Ebro has been explained in detail in Section 3.1.4. As already mentioned, the inventories of several earlier pieces of research on the production process of RCA-CDWs will be taken into account in order to compare them with the production of 1 tonne of RCA-CBWs.

- LCI Hormigones Ebro

The recycling process of the CBWs (see Figure 3) does not involve the consumption of large consumables except for the diesel used in the aggregate crushing machine. The crushing machine has an average production rate of 200 t/h, and its diesel consumption at maximum output is 67.8 L/h. This gives a diesel consumption of approximately 0.34 L per tonne of RCA-CBWs produced. This value is in line with the interval values proposed by ANEFA for the production of RCA-CDWs with diesel with a value of 0.89 ± 0.44 L [17]. Diesel fuel has an energy of 10.96 kWh/L which results in an energy consumption of 3715 kWh for the production process of the RCA-CBWs. The inventory is shown in Table 3. This RCA-CBWs production process has zero water consumption. It should be noted that the grinding of the CBWs leads to wear of the steel grinding wheels and these have to be replaced from time to time if it is decided to include them in the inventory. For this purpose, and due to a lack of specific information on this consumable, we decided to choose the value proposed by ANEFA for this consumable, shown in Table 4.

- LCI RCA-CDWs

This section describes the inventories made in the existing literature on the production of RCA-CDWs. Generally, research that conducts an environmental assessment of the production of RCA-CDWs has established its own inventories. These inventories vary due to the degree of detail required for the research. For example, some research considers the steel costs of the crushing process or the tire costs of machines such as the excavators that load the waste into the feeding chutes. However, all the works share a number of fundamental common consumptions, such as electricity, diesel fuel and water. Detail is provided in Table 4, the inventory of consumption established by ANEFA [17]. We assume that the screen meshes and crushing rollers used are made of low alloy steel, and therefore, they are modelled in the SimaPro software with the same process.

With regard to the existing literature, a wide range of previous studies assess the sustainability of the use of RCAs in specific cases [9,24,28,29]. These investigations logically contain their own inventory (with a greater or lesser degree of detail) and, therefore, also their respective results derived from applying the various environmental methodologies available. Therefore, in order to contrast previous results with those obtained in this research, we decided to choose two impact categories that are present in the vast majority of environmental assessment methodologies. These are the global warming potential (unit: kg of CO_2_) and the use of non-renewable primary energy (unit: MJ), i.e., the consumption of energy from sources such as fossil fuels.

- Transport

In the transport process, the movement of the RCAs and other raw materials from the place of manufacture to the point of use, mixing or recycling is included. The aim of studying this process is to model particulate emissions, gases such as nitrogen oxides (NO_x_) and carbon oxides (CO_x_), which are produced as a result of fuel combustion in the internal combustion engine of the means of transport and which logically have an impact on the environment. The transport was modelled on a 32 metric tonne capacity truck using diesel fuel, to which the European EURO 6 emission standard is applied [32]. It was decided to choose a transport process that meets these pollutant standards in order to make it more realistic, as nowadays European directives increasingly restrict pollution in transport. The process chosen in SimaPro is as follows: Transport, freight, lorry >32 metric ton, EURO 6 {RER}|Cut-off, U (Ecoinvent v.3) and its defined unit is metric tonne per km (mt-km). From here, it is a matter of defining average distances or distances that are known directly from the literature. These transport distances are for the transport of cement, fine aggregates, coarse aggregates and recycled aggregates.

Cement is the material that travels the longest average distance between its place of origin to its point of use. In Spain, this distance varies between 50~400 km, according to research by Fraga et al. (2014) [33]. We decided to choose an average distance of 200 km. Consequently, the transport value associated with cement will be 60 mt-km.

This transport distance is reduced for the transport of fine aggregates from the quarry where they are produced and definitively treated to the place of use. In this study, where we know the exact locations of both the quarry and the point of manufacture of the concrete samples, the distance is 15 km. This falls within the range of average distances established by Fraga et al. (2014) for aggregates (15~20 km) [33]. In Spain, these reduced distances constitute an advantage in favour of the use of NAs instead of RAs. However, in countries with a shortage of NA production or excessive transport distances resulting in high economic costs, the use of recycled aggregates would be more advantageous.

No transport process is applied to the RCA-CBWs because they are produced and used on-site in the same concrete production plant, and the distance they are transported is minimal. This is an advantage compared to RCA-CDWs produced in a fixed recycling plant (Figure 4), which have to make at least two trips. The first journey is from the CDW collection site to the recycling plant where the RCA-CDWs are produced, and the second is the transport of the RCA-CDWs to the implementation site. These distances can vary greatly and depend on the case study, as can be seen in Table 5 where the distances proposed by other studies for the transport of CDWs to a fixed recycling plant are shown.

As can be seen, the production of RCA-CDWs involves the addition of a transport process that does not exist for the case study in this research. This lends an advantage to the RCA-CBWs under study in this research, as explained in the results sections.

- Production of the concrete samples

The samples must be prepared in concrete or mortar production plants. These plants have sufficient room to stockpile fine and coarse aggregates and cement. Generally, these production plants have a very low level of machinery, limited to mixing machinery, dosing machines, conveyor belts, etc. The energy consumed by these plants is electrical. The research by Fraga et al. (2014), calculates the electrical energy required to produce 1 m^3^ of ready-mixed concrete at 1.61 kWh/m^3^ [33]. To model this process, we chose an existing concrete production process in SimaPro: Concrete, 25–30 MPa {RoW} concrete production 25–30 MPa|Cut-off, U (Ecoinvent v.3). The electricity consumption in the process was modified to the actual values in this research and for electricity from the national medium voltage grid (Electricity, medium voltage {ES} market for|Cut-off, U—Ecoinvent v.3).

#### 3.1.5. Life Cycle Impact Assessment (LCIA)

This stage is the final stage before the interpretation of the results. It involves attributing the various inflows and outflows identified in the LCI to impact categories (phase-classification). The potential impacts are also quantified (phase-characterisation) using various impact assessment methods/methodologies [13]. The catalogue of environmental methodologies is hugely diverse (CML Baseline, Cumulative Energy Demand, EPD, Eco-Indicator, ReCiPe, etc.) [28]. They differ in the number of impact categories they assess, which can be very specific or more general. In addition, each methodology performs a different classification and characterisation process, which means different results can be obtained for the same study. For this research, the EPD 2018 methodology, named after its acronym environmental product declaration (EPD), was chosen. This methodology takes into account the impact categories set out in Table 6. It is often used to assess products within the construction sector as it allows for differentiation of environmental impacts for different products [5].

### 3.2. Economic Analysis

This section aims to study the economic cost of using RCA-CBWs in concrete and to determine their economic viability compared to concrete made with naturally produced aggregates. To do so, the prices of the raw materials used in the concrete must be established. We assumed that the prices of the raw materials shown in this section take into account the direct production costs (transport, raw materials used in their manufacturing process, labour etc.). This assumption intends to avoid complexity in the economic data analysis. Table 7 shows the raw material prices used in the existing research [10,24,29]. As can be seen, there is a big difference between the prices used in the different studies. There is less difference between the prices established for aggregates (fine and coarse) in the research by Braga et al. (2017) [29] and Dias et al. (2021) [24]. This is because these prices only include the costs associated with production activities (striping, blasting, sorting and crushing) [24]. However, in the research by López Ruiz et al. (2022) [10], the costs attributed to the aggregates are higher because they include the transport process and environmental costs (air emissions, water emissions, landfill charges) [10]. The prices used in the research are taken from in-house data from companies in the construction sector.

The variability in the price of the aggregates is also related to geographical location. For example, the findings of Cerchione et al. (2023) indicate that in aggregate-scarce countries such as the UK and Belgium, the cost of aggregates increases significantly to 25€/t in the UK and 35€/t in Belgium. Thus, the same pattern is seen with RAs, which range from 2–6€/t in countries such as Portugal, Spain and Italy, compared to 12–15€/t in countries such as the UK and Belgium. One advantage in these countries is that, although they have low levels of NA production, the landfill fees for CDW are very high (100€/t in the UK, 125€/t in Belgium) compared to Italy, for example (1–10€/t) [13]. This remarkable and visual difference means that in countries with fewer NA resources, RAs are economically more viable and, therefore, the construction sector is more interested in using them. This situation contrasts with that seen in countries where the exploitation of NAs is high due to their availability, which means that, from an economic point of view, the production of NAs is more viable and profitable. Until there is a change of trend in favour of RAs supported by public policy, the use of NAs will remain predominant.

## 4. Results and Discussion

### 4.1. Results of the Production of 1 m^3^ of Concrete from the Samples

**Figure 5:**
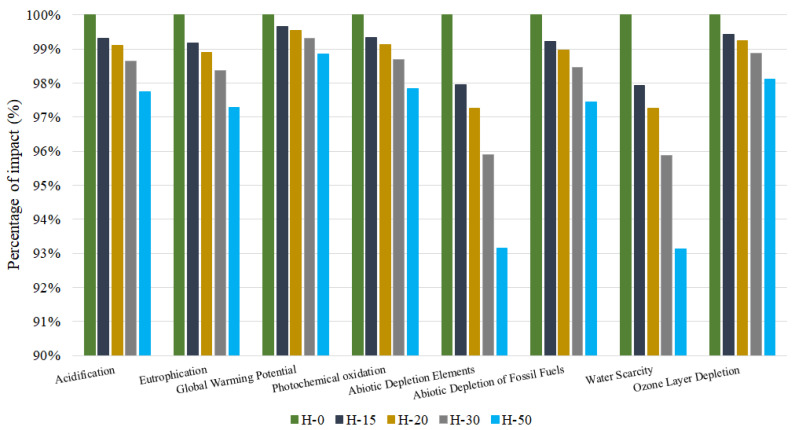
Environmental results for the production of 1 m^3^ of concrete from the samples tested.

As shown in Figure 5, the environmental results are given for the production of the various 1 m^3^ concrete samples. The *y*-axis shows the percentage of impact of each sample for each impact category (*x*-axis). For enhanced visualisation, only the last 10% of the impact is shown, i.e., from 90% to 100%. In all the impact categories, the graph shows a downward trend from the baseline concrete sample H-0 to the H-50 sample containing RCA-CBWs. Overall, this implies that the higher the percentage of NCAs substituted for RCA-CBWs, the lower the environmental impacts of producing 1 m^3^ of concrete.

In more detail, the abiotic depletion elements category shows a 6.84% decrease in the percentage of impact. Evidently, this category will see a decrease in the impact generated as the use of RCA-CBWs means that virgin natural resources such as the minerals that make up the NCAs will not be used. The benefits of not producing NAs are also evident in the water scarcity category, with a reduction of 6.87%. It is well known that the production of NAs involves the use of water for internal washing and sieving processes and the creation of dust. The H-50 sample achieves a saving of 4362 m^3^ of water for every cubic metre of concrete produced. These water savings values are highly promising, since in the future this resource may be affected by severe droughts and therefore revalued. Moreover, these savings will increase exponentially for large quantities of concrete used. Both of these categories, abiotic depletion elements and water scarcity, are related to the impact category land use. The use of RCA-CBWs instead of NCAs avoids any impact on land use. This is because it prevents the creation of new areas for the NCAs extraction and the mining industry. By avoiding the destruction of vegetation cover and the geography of the land through blasting, which will indirectly affect impact categories such as acidification, eutrophication, ozone layer depletion and photochemical oxidation, as will be seen later.

Other categories such as ADFF (−2.55%) and Ozone Layer Depletion (−1.89%) also see an improvement in their impacts. These categories best represent the process of transporting NAs from the quarry to the concrete production plant. The transport process emits harmful particulate matter into the atmosphere, as well as greenhouse gases (CO_x_, NO_x_), mainly due to the burning of fossil fuels such as diesel. These decreases are clearly demonstrated in the H-50 sample, which avoids the production-transport of 300 kg of NCAs. The other categories evaluated such as acidification (−2.25%), eutrophication (−2.72%), photochemical oxidation (−2.17%) and GWP (−1.14%) also reveal environmental benefits, although to a lesser extent. The most striking of these is the GWP impact category, where the H-50 sample achieves a net emission reduction of 3.42 kg of CO_2_/m^3^ compared to the H-0 sample.

One of the most notable findings in favour of the RCA-CBWs obtained in this research is that the higher the percentage of NCAs substituted by RCA-CBWs the lower the environmental impacts generated. This is contrary to the literature [8], which says that impact categories related to energy use and emissions of harmful substances into the atmosphere increase when using RCA-CBWs. The explanation for this difference is that, in this study, the RCA-CBWs are produced in the concrete production plant itself. This avoids the need for transport and, therefore, its consequent impacts, which will be explained in more detail below.

**Figure 6:**
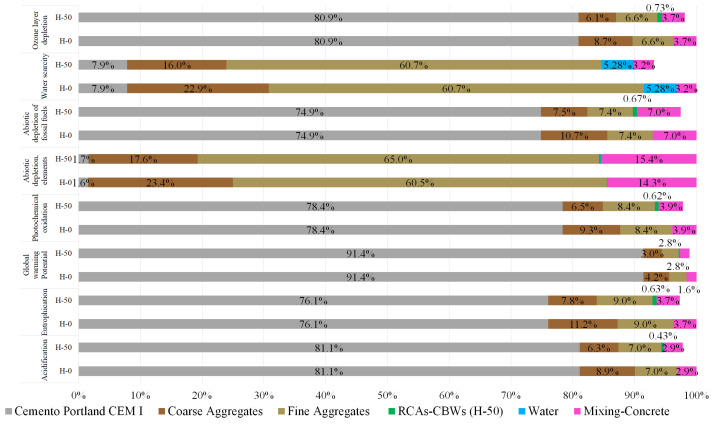
Comparison of results by raw materials for samples H-0 and H-50.

Given the overall environmental benefits of using the RCA-CBWs studied over the use of NCAs, we decided to illustrate the results obtained in each impact category for each of the raw materials in the sample. In other words, to visually represent the impact load generated by cement, fine aggregates, coarse aggregates, RCA-CBWs, water and the mixing process. The results are shown in Figure 6 where the mixtures of H-0 and H-50 are contrasted. As can be seen in Figure 6, the H-50 mixture does not reach 100% of the environmental impacts in the evaluated categories, it is justified because it is being compared with the H-0 mixture. It is in this H-0 mixture where the greatest environmental impacts occur, and therefore, considering it as the reference mixture, it is assigned the percentage of 100%.

As the graph shows, cement generates the biggest environmental impacts by far, regardless of the sample assessed. For example, for the GWP impact category its impact percentage is 91.4%. This means that for every 1 m^3^ of H-0 concrete produced, of the 300.41 kg of total CO_2_ emitted, 274.67 kg of CO_2_ is contributed by the cement. According to López et al. (2010), this is due to the high energy consumption and the respective emissions from the calcination reaction in the cement kiln [10]. The second biggest contributor to the impacts is the production of NAs (fine and coarse). When tested for the H-0 sample, natural fine aggregates have a more significant negative impact than coarse aggregates in certain categories. The most notable differences are in the categories of water scarcity (fine aggregates 60.73% and coarse aggregates 22.9%) and in the category of abiotic depletion elements (fine aggregates 60.54% and coarse aggregates 23.44%). This significant difference in impact is caused because the production of fine aggregates involves more steps than those required to produce coarse aggregates, such as more crushing to achieve the desired particle size and more intricate washing and sieving processes. By contrast, in other categories, coarse aggregates have greater impact, but with a reduced margin of difference compared to fine aggregates. These are acidification (1.97%), eutrophication (2.13%), GWP (1.45%), photochemical oxidation (0.89%), abiotic depletion elements (3.38%), and ozone layer depletion (2.09%). The difference can be explained considering that the total amount of coarse aggregates (6/12 and 12/20) is higher than that of fine aggregates (0/6).

Lastly, if we focus the results to show the differences obtained from the simulation between NCAs and RCA-CBWs, they yield positive data. Firstly, let’s consider the results for the RCA-CBWs production process. As examined in Figure 6, the study’s modelling shows that the impact of producing RCA-CBWs is minimal, being most notable at 0.73% for ozone layer depletion, 0.67% for ADFF and 0.62% for photochemical oxidation. Clearly, the categories with the most significant impact are those related to the use of diesel fuel and its use in the CBW crushing engine, which is the only resource used in the production process of the RCA-CBWs. If these RCA-CBWs had undergone prior transport, these categories would undoubtedly have seen their impacts increase. However, when studying the production of RCA-CBWs excluding transport due to the minimal distance between their production and usage sites, the environmental benefits of their use are significantly increased. Having commented on the results for the RCA-CBWs production process, we can move on to consider the differences between the impacts of the NCAs in the H-0 and H-50 samples. Naturally, replacing the NCAs with RCA-CBWs results in a reduced impact because there is a 300 kg saving in NCAs between samples H-0 and H-50. The average reduction for all impact categories is 8.43%, increasing to differences of 17.41% for abiotic depletion elements and 16.03% for water scarcity. It should be noted that if the average impact reduction percentage of 8.43% associated with producing NCAs were lower than the average impact percentage associated with producing the RCA-CBWs, they would be environmentally unfeasible because the use of RCA-CBWs would imply greater environmental impacts than the use of NCAs. For other impact categories, the reductions achieved by using RCA-CBWs instead of NCAs are acidification (−5.82%), eutrophication (−7.19%), GWP (−2.83%), photochemical oxidation (−5.89%), abiotic depletion elements (−6.85%), and ozone layer depletion (−5.38%).

In summary, this first part of the results demonstrates the environmental viability of replacing NCAs (12/20) with RCA-CBWs (12/20), saving 4.36 m^3^ of water per 1 m^3^ of concrete, avoiding the consumption of 37.57 MJ/m^3^ of fossil fuels and avoiding the emission of 3.42 kg of CO_2_/m^3^ into the atmosphere.

### 4.2. Results for the Production of 1 Tonne of RCAs

**Figure 7:**
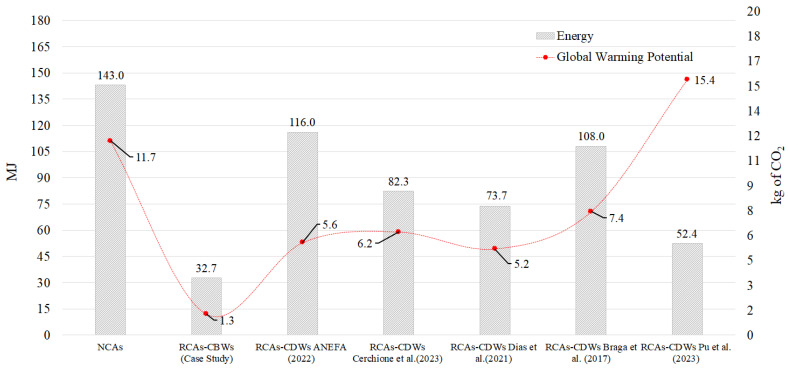
Comparison between the production of 1 tonne of RCAs and the findings of previous research. ANEFA (2022) [17], Cerchione et al. [13], Dias et al. [24], Braga et al. [29], Pu et al. [14].

Figure 7 shows the environmental results obtained by comparing the production of 1 tonne of RCA-CBWs with the existing literature (RCA-CDWs), in order to determine the differences between the processes and to see how they affect the utilisation of these RCA-CBWs. Figure 7 contains a double-axis graph. In this graph, the left-hand *y*-axis indicates the amount of energy in MJ used to produce 1000 kg of RCAs, while the right-hand *y*-axis shows the emissions of kg of CO_2_ emitted during the production of the RCAs.

In this study, the results for the production of 1 tonne of RCA-CBWs were the most positive environmental results obtained. The process only emits 1.3 kg of CO_2_/t and consumes 32.7 MJ/t of energy. The process of producing RCA-CBWs only requires a small amount of diesel fuel for the operation of the CBW crusher engine; therefore; this reduced fuel consumption leads to the previously mentioned results. This is in comparison to the research conducted by Cerchione et al. (2023), where the energy consumed to produce 1 tonne of RCA-CDWs was 60.31% more, translating to 49.62 MJ. The CO_2_ emissions were also higher, by 79.53%, equivalent to emitting 4.95 kg of CO_2_ more per tonne of RCA-CDWs [13]. These considerable differences in the results are due to the fact that in the Cerchione et al. (2023) research, the CDWs had to be transported from the demolition site to be converted into RCAs at the recycling plant. This is reflected in their research, where, when analysing the environmental impacts of producing 1 tonne of RCA-CDWs, the transport process reaches average values of 80% in the impact categories [13]. Evidently, reducing the need to transport virgin waste for the production of RCAs helps to mitigate the impacts considerably. These findings are corroborated by the research of Braga et al. (2017) [29].

In the research conducted by Dias et al. (2021) [24], the relevance of transporting CDWs prior to the production of RCA-CDWs and its effects on the assessed environmental impact categories can be clearly identified [24]. For example, the total CO_2_ emissions associated with producing 1 tonne of RCA-CDWs is 5.2 kg. Dias et al. (2021) [24] identify that this net CO_2_ emissions value is subdivided into a value of 2.5 kg of CO_2_ emitted during the internal production process and 2.7 kg of CO_2_ generated in the process of transporting the CDWs from the demolition site to the recycling plant. This implies that 51.9% of emissions are transport related. The same is true for energy consumed, whereby 50% of it is used in the transport process [24]. Evidently, the results obtained are for a specific case study. However, the conclusions, extrapolated to a general framework, are that the further the CDWs have to be transported, the more unfeasible their use.

When analysing the production of 1 tonne of NCAs, it is important to remember that the results only account for the internal transport process in the quarry. They do not account for transporting the NCAs from the quarry to a concrete production plant (15~20 km) [33]. As can be seen, the production of these NCAs emits 11.7 kg of CO_2_. These emissions are only surpassed in the research conducted by Pu et al. (2023) [14], who assessed emissions of 15.4 kg of CO_2_. Furthermore, a comparison of the MJ of energy consumed shows that the RCA production processes of the studies that were analysed uses less energy than the process of producing 1 tonne of NCAs according to the process modelled in SimaPro.

Finally, the results obtained by ANEFA for the production of 1 tonne of RCA-CDWs [17] are quite similar to those obtained in other existing research works. For example, CO_2_ emissions are 5.6 kg of CO_2_/t, a value very similar to that of research works [13,24,29]. Interestingly, however, the energy consumption is somewhat higher. This is because ANEFA’s process inventory for RCA-CDWs is derived from average data for production facilities of RCA-CDWs. In other words, it uses an RCA-CDW production model that uses energy sources such as diesel and electricity, whereas, in reality, an RCA-CDW production plant does not have to consume both energy sources. The same would be true for water consumption. Incorporating all these elements into the inventory increases the environmental impacts and, in the case under analysis, energy and CO_2_ emissions. Despite this drawback, it should be noted that the results are within an acceptable range and are therefore reliable.

### 4.3. Economic Results

**Figure 8:**
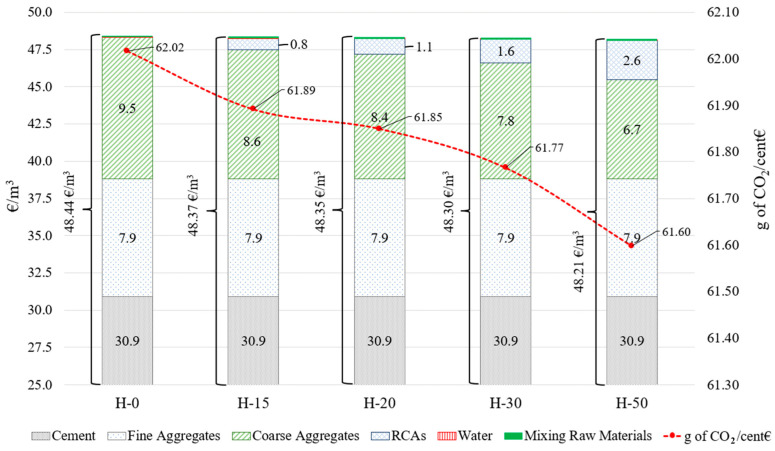
Economic analysis of the samples evaluated.

Figure 8 shows the results of the economic analysis of the various samples studied. It is worth pointing out that the higher the quantity of RCA-CBWs, the lower the economic cost per cubic metre of concrete. This conclusion is also drawn by Braga et al. (2017), who indicate that concrete made with RCA-CDWs is cheaper than concrete made with NCAs [29]. The reduction, however, is almost negligible. Nevertheless, even if the differences are very small, as will be explained in detail below, they reflect an important overall finding: the use of RCA-CBWs does not increase the cost of producing the same volume of concrete. Therefore, reusing CBWs as RCAs is economically viable and provides an opportunity to recycle a waste product that would traditionally have been landfilled without any kind of treatment.

If the results are analysed in detail, the most significant difference is found between the H-0 sample and the H-50 sample, with a price decrease of −23€cents/m^3^ (−0.46%). These differences are minimal because firstly, the difference between the economic cost of NCAs and RCA-CBWs based on the prices used in this research is 0.8€/tonne. Secondly, the unit of volume used is 1 m^3^ of concrete is too little to observe economically significant differences.

Lastly, the columns show the price broken down by raw materials for 1 m^3^ of concrete. In order to illustrate the findings more clearly, only the last €25 of the price is shown on the ordinate axis. The highest economic cost is associated with the use of the raw material cement [29], although this cost (30.9€/m^3^) remains constant for all the samples analysed, as does the cost of fine aggregate (7.9€/m^3^), water (0.3€cent/m^3^) and, lastly, the mixing process of the raw materials (0.12€/m^3^). The comparison of coarse aggregates helps to better understand the economic benefit. As shown for alternative H-0, the NCAs have a cost of 9.5€/m^3^, representing 19.61% of the total per m^3^. It is the second most significant economic cost after the cement (63.79%). By contrast, in 1 m^3^ unit of concrete, the costs of water consumption or the mixing process itself are irrelevant, as the sum of both costs only accounts for 0.25% of the total. As the RCA-CBW percentages increase in the different samples, the total costs (€/m^3^) are reduced. For example, alternatives H-0 and H-50 have a 2.85€/m^3^ price difference and a 300 kg difference in the mass of NCAs. However, producing the RCAs also comes at a cost and, as above, implies a difference in price between the most extreme alternatives of H-0 and H-50, which, in this case is 2.6€/m^3^. It is important to note that, according to our data, producing RCA-CBWs is −0.8€/t cheaper than producing NCAs. If this price difference were positive (i.e., increasing the cost), it would be more economically feasible to use NCAs instead of RCA-CBWs. According to the data obtained, we can define a net economic decrease factor in favour of using RCA-CBWs of −0.075€cent per kg of RCA-CBWs used. Logically, this calculated factor will depend on the retail price of RCAs in the country of use. This is because, in other countries, as discussed above, the price of RCAs may be higher than that of NCAs. Therefore, it generates a higher economic cost per m^3^ of concrete, and will be a disadvantage in favour of the use of NCAs instead of RCAs.

Another indicator we wanted to highlight, which we will call an emission cost comparison, is the emissions in kg of CO_2_ emitted for each monetary unit of cost per cubic metre of concrete. In this case, the currency unit is the euro (€). This indicator is plotted in Figure 8 with a red dotted line. As can be seen, it has a negative downward slope. The interpretation of this indicator is that the higher the level of RCA-CBWs in the samples, the lower the emission in kg of CO_2_ emitted for each euro (€) of the cost of the cubic metre of concrete. As before, the most significant differences occur between the H-0 and H-50 alternatives, with a difference in value of −0.420 g of CO_2_/€cent. This indicator could be helpful in the future for evaluating the benefits of using different types of concrete on both an environmental and economic level.

## 5. Conclusions

This research has demonstrated the environmental and economic viability of using waste generated in a concrete plant, such as RCA-CBWs. By giving CBWs a new reuse pathway and integrating them back into the value chain through the application of circular economy criteria.

In environmental terms, the existing benefits of using RCA-CBWs instead of traditional NCAs are clearly evident. These environmental benefits increase as the percentage of replacement of NCAs by RCA-CBWs increases. The largest differences are reflected for substitution percentages of 50%. For this specific case and on average, the environmental impacts are reduced by −3.30% for all impact categories. However, significant decreases in impacts stand out in categories such as water scarcity, with a reduction of −6.87% (−4.36 m^3^ of water/m^3^), and abiotic depletion elements, with a decrease of −6.84% (−8.483–10^−5^ kg Sb eq/m^3^). Another great results to highlight is that environmental benefits are shown in categories such as ADFF, with a reduction of −2.55% (−37.57 MJ/m^3^) and GWP, with −1.14% (−3.419 kg of CO_2_/kg). These impact categories were previously reported by other research to have increased impacts when using RCA-CDWs instead of NCAs.

In economic terms, it is found that using RCA-CBWs in the evaluated concrete samples does not increase the economic cost, which is a positive outcome in favour of using this type of aggregate. In fact, using RCA-CBWs implies a reduction in the economic cost, albeit a very minor one of −0.46% (−0.23€/m^3^). There is calculated to be an economic decrease factor of −0.075€cent/kg of RCA-CBWs, which shows the decreasing economic burden of using RCA-CBWs per unit of mass. Finally, the environmental study and the economic study are related through the emission cost factor which has a value of −0.42 g of CO_2_/cent€, showing that the alternatives that incorporate RCA-CBWs emit less CO_2_ for each financial unit of the production cost.

It should be mentioned that the use of RCA-CBWs in concrete results in a slight decrease in mechanical outcomes compared to the use of NCAs, limiting the range of application of RCA-CBWs to the construction sector, for instance for non-structural applications. It is hoped that this research will provide new results and information on using RAs instead of NAs, consequently furthering knowledge in this area of the construction sector and helping it to achieve sustainability and a circular economy in the future.

## Figures and Tables

**Table 1:** Samples evaluated in 1 m^3^ of ready-mixed concrete.

Material	Designation Substitution Percentage	H-0	H-15	H-20	H-30	H-50
		0%	15%	20%	30%	50%
Cement	CEM I-32.5R (kg)	300				
Natural Fine Aggregates	FA-0/6-T-S-L (kg)	833.3				
Natural Coarse Aggregates	CA-6/12-T-S-L (kg)	400				
	CA-12/20-T-R (kg)	600	510	480	420	300
RCAs	CA-12/20-T-R (kg)	-	90	120	180	300
Water	Water (kg)	163				

**Table 2:** Inventory of the materials comprising the samples and their process definition in SimaPro 9.2.0.2.

Material	Unit	H-0	H-15	H-20	H-30	H-50	SimaPro Process
Cement	kg	300					Portland cement (CEM I), CEMBUREAU technology mix, CEMBUREAU production mix, at plant. (ELCD)
Fine Aggregates 0/6	kg	833.3					Sand {RoW} market for sand|Cut-off, U. (Ecoinvent v.3)
Coarse Aggregates 6/12	kg	400					Gravel, crushed {RoW} market for gravel, crushed|Cut-off, U. (Ecoinvent v.3)
Coarse Aggregates 12/20	kg	600	510	480	420	300	
RCA-CBWs 12/20	kg	-	90	120	180	300	CBWs Recycling Process
Water	kg	163					Tap water {RER} market group for|Cut-off, U. (Ecoinvent v.3)

**Table 3:** LCI for the production of RCA-CBWs.

Material	Unit	Amount	SimaPro Process
Diesel Fuel	L	0.334	Diesel {RER} market group for|Cut-off, U
Combustion Diesel Shredder	kWh	3.715	Diesel, burned in building machine {GLO}|Cut-off, U
Steel Shredder	t	6.9·10^−6^	Steel, low alloyed {GLO} market for|Cut-off, U

**Table 4:** LCI for the production process of 1 tonne of RCA-CDWs according to ANEFA [17].

Material	Unit	Amount	SimaPro Process
Diesel Fuel	L	0.89	Diesel {RER} market group for|Cut-off, U
Combustion Diesel Fuel	kWh	9.75	Diesel, burned in building machine {GLO}| Cut-off, U
Electricity	kWh	2.22	Electricity, medium voltage {ES} market for|Cut-off, U
Transport	mt·km	22.68	Transport, freight, lorry >32 metric ton, EURO 6 {RER}|Cut-off, U
Water	m^3^	0.05	Tap water {RER} market group for|Cut-off, U
Tires	t	2.2168·10^−5^	Waste, pneumatic tyres {GLO} market for waste, pneumatic tyres|Cut-off, U
Screening mesh	t	2.40828·10^−6^	Steel, low alloyed {GLO} market for|Cut-off, U
Rollers	t	4.49246·10^−6^	

**Table 5:** Distances evaluated in existing literature on the transport of CDWs to a fixed recycling plant.

Research	Transport Distance of CDWs
Cerchione et al. (2023) [13]	30.0 km
Pu et al. (2023) [14]	20.0 km
López Ruiz et al. (2022) [10]	18.0 km
Colangelo et al. (2020) [26]	30.0 km
Gayarre et al. (2016) [8]	40.0 km

**Table 6:** Impact categories in the EPD-2018 methodology.

Impact Categories	Unit
Acidification	kg SO_2_ eq
Eutrophication	kg PO_4_ eq
Global Warming Potential (GWP)	kg CO_2_ eq
Photochemical Oxidation	kg NMVOC
Abiotic Depletion Elements	kg Sb eq
Abiotic Depletion of Fossil Fuels (ADFF)	MJ
Water Scarcity	m^3^
Ozone Layer Depletion	kg CFC-11 eq

**Table 7:** Average price of raw materials used for the production of the samples assessed.

Material	Case Study	López Ruiz et al. (2022) [10]	Braga et al. (2017) [29]	Dias et al. (2021) [24]
Cement	103 (€/t)	99.37 (€/t)	90 (€/t)	-
Fine Aggregates	9.50 (€/t)	17.46 (€/t)	4.15 (€/t)	4.60 (€/t)
Coarse Aggregates		18.37 (€/t)	4.59 (€/t)	
RCAs	8.90 (€/t)	11.00 (€/t)	2.00 (€/t)	2.00 (€/t)
Water	0.02 (€/m^3^)	1.60 (€/m^3^)	1.53 (€/m^3^)	-
Mixing of materials	0.12 (€/m^3^)	11.40 (€/m^3^)	-	-

## Data Availability

Data are contained within the article.

## References

[1] Ricciotti L., Occhicone A., Ferone C., Cioffi R., Roviello G. (2024). Eco-design of geopolymer-based materials recycling porcelain stoneware wastes: A life cycle assessment study. Environ. Dev. Sustain..

[2] Código Estructural Minsiterio de Transportes, Movilidad y Agenda Urbana. https://www.codigotecnico.org/DocumentosCTE/SeguridadEstructural.html.

[3] Ferreiro-Cabello J., Fraile-Garcia E., Pernia-Espinoza A., Martinez-de-Pison F.J. (2022). Strength Performance of different mortars dopes using olive stones as lightweight aggregate. Buildings.

[4] Sodupe-Ortega E., Fraile-Garcia E., Ferreiro-Cabello J., Sanz-Garcia A. (2016). Evaluation of crumb rubber as aggregate for automated manufacturing of rubberized long hollow blocks and bricks. Constr. Build. Mater..

[5] Los Santos-Ortega J., Fraile-García E., Ferreiro-Cabello J., González-González C. (2023). Mechanical and Environmental Assessment of Lathe Waste as an Addiction to Concrete Compared to the Use of Commercial Fibres. Materials.

[6] Chen J., Yu J., Nong Y., Yang Y., Zhang H., Tang Y. (2023). Beyond time: Enhancing corrosion resistance of geopolymer concrete and BFRP bars in seawater. Comps. Struct..

[7] Tang Y., Wang Y., Wu D., Liu Z., Zhang H., Zhu M., Chen Z., Sun J., Wang X. (2022). An experimental investigation and machine learning-based prediction for seismic performance of steel tubular column filled with recycled aggregate concrete. Adv. Mater. Sci..

[8] Gayarre F.L., Pérez J.G., Pérez C.L.C., López M.S., Martínez A.L. (2016). Life cycle assessment for concrete kerbs manufactured with recycled aggregates. J. Clean. Prod..

[9] Iodice S., Garbarino E., Cerreta M., Tonini D. (2021). Sustainability assessment of Construction and Demolition Waste management applied to an Italian case. Waste Manag..

[10] Ruiz L.A.L., Ramon X.R., Mercedes C.M.L., Domingo S.G. (2022). Multicriteria analysis of the environmental and economic performance of circularity strategies for concrete waste recycling in Spain. Waste Manag..

[11] Blengini G.A., Garbarino E. (2010). Resources and waste management in Turin (Italy): The role of recycled aggregates in the sustainable supply mix. J. Clean. Prod..

[12] Borghi G., Pantini S., Rigamont L. (2018). Life cycle assessment of non-hazardous Construction and Demolition Waste (CDW) management in Lombardy Region (Italy). J. Clean. Prod..

[13] Cerchione R., Colangelo F., Farina I., Ghisellini P., Passaro R., Ulgiati S. (2023). Life Cycle Assessment of Concrete Production within a Circular Economy Perspective. Sustainability.

[14] Pu Y., Li L., Shi X., Wang Q., Abomohra A. (2023). A comparative life cycle assessment on recycled concrete aggregates modified by accelerated carbonation treatment and traditional methods. Waste Manag..

[15] Los Santos-Ortega J., Fraile-García E., Ferreiro-Cabello J. (2023). Methodology for the environmental analysis of mortar doped with crumb rubber from end-of-life tires. Constr. Build. Mater..

[16] Fraile-Garcia E., Ferreiro-Cabello J., López-Ochoa L.M., López-González L.M. (2017). Study of the Technical Feasibility of Increasing the Amount of Recycled Concrete Waste Used in Ready-Mix Concrete Production. Materials.

[17] Asociación Nacional de Empresarios Fabricantes de Áridos (ANEFA) Áridos Sostenibles. Indicadores Sectoriales. https://aridos.info/wp-content/uploads/2022/05/InformeAmbiental.pdf.

[18] Cuesta V.R., López V.O., Skaf M., Fiol F., Manso J.M. (2022). Why is the effect of recycled concrete aggregate on the compressive strength of self-compacting concrete not homogeneous? A bibliographic review. Inf. Constr..

[19] Trinchese G., Verniero A., Osa G.G.L. (2022). New recycling technologies of demolished materials for sustainabe finishes: The project of concrete reuse on site in Tres Cantos, Madrid. VITRUVIO-Int. J. Archit. Technol. Sustain..

[20] (2011). Cement. Part I.

[21] (2006). Environmental Management—Life Cycle Assessment—Principles and Framework.

[22] (2006). Environmental Management—Life Cycle Assessment—Requirements and Guidelines.

[23] Pesta J., Pavlu T., Koci V. (2019). Life Cycle Assessment of Recycling Processes for Demolition Waste. IOP Conference Series: Earth and Environmental Sciences (EES) Provides a Fast, Versatile and Cost-Effective Proceedings Publication Service.

[24] Dias A.B., Pacheco J.N., Silvestre J.D., Martins I.M., de Brito J. (2021). Environmental and economic life cycle assessment of recycled coarse aggregates: A Portuguese case study. Materials.

[25] Colangelo F., Forcina A., Farina I., Petrillo A. (2018). Life Cycle Assessment (LCA) of different kinds of concrete containing waste for sustainable construction. Buildings.

[26] Colangelo F., Navarro T.G., Farina I., Petrillo A. (2020). Comparative LCA of concrete with recycled aggregates: A circular economy mindset in Europe. Int. J. Life Cycle Assess..

[27] Atta I., Bakhoum E.S. (2023). Environmental feasibility of recycling construction and demolition waste. Int. J. Environ. Sci. Technol..

[28] Dias A. (2022). Environmental and Economic Comparison of Natural and Recycled Aggregates Using LCA. Recycling.

[29] Braga A.M., Silvestre J.D., Brito J. (2017). Compared environmental and economic impact from cradle to gate of concrete with natural and recycled coarse aggregates. J. Clean. Prod..

[30] Ecoinvent v3.0. https://ecoinvent.org.

[31] European Platform on Life Cycle Assessment-European Commission. https://data.jrc.ec.europa.eu/collection/EPLCA.

[32] Emisiones de los Vehículos Pesados (Euro VI). https://eur-lex.europa.eu/ES/legal-content/summary/emissions-from-heavy-duty-vehicles-euro-vi-certification-rules.html.

[33] Fraga J.M., Gochi A.D.C., López M.P.D.L.C. Sostenibilidad en la preparación y puesta en obra de hormigón en España: Análisis de consumo energético y emisiones de CO_2_. Proceedings of the 18th International Congress on Project Management and Engineering.

